# Extrahepatic portal venous obstruction: The effects of early ligation of splenic artery during splenectomy

**DOI:** 10.4103/0971-9261.59600

**Published:** 2009

**Authors:** Suhasini Gazula, D. K. Pawar, T. Seth, C. S. Bal, V. Bhatnagar

**Affiliations:** Department of Pediatric Surgery, All India Institute of Medical Sciences, New Delhi, India; 1 Department of Anaesthesiology, All India Institute of Medical Sciences, New Delhi, India; 2 Department of Hematology, All India Institute of Medical Sciences, New Delhi, India; 3 Department of Nuclear Medicine, All India Institute of Medical Sciences, New Delhi, India

**Keywords:** Extra hepatic portal venous obstruction, hypersplenism, platelets, red blood cells, splenectomy, transfusion

## Abstract

**Aim:**

To objectively demonstrate the gain in blood volume and blood components following early ligation of splenic artery during splenectomy and splenorenal shunts in children with extra hepatic portal venous obstruction (EHPVO).

**Methods:**

Twenty-eight children (20 males and 8 females, mean age: 9.9 (±3.2) years) with EHPVO and hypersplenism were recruited. We followed a protocol of systematically locating and ligating the splenic artery first, followed by a 30-minute waiting period to allow the massive spleen to decongest via the splenic vein and venous collaterals and then completing the splenectomy by standard procedure. No intravenous fluid was administered during this 30-minute period. Blood samples were drawn just prior to splenic artery ligation and soon after splenectomy for the estimation of hematological and biochemical parameters.

**Results:**

We noticed a highly significant increase in the hemoglobin, hematocrit, leukocyte, platelet, and RBC counts by early ligation of the splenic artery (p < 0.0004). The gain in hemoglobin and hematocrit was equivalent to a transfusion of atleast 100-150 ml of packed RBC. The increase in platelet count was equivalent to a platelet transfusion of atleast 4 units of platelet concentrates in an adult. There is a positive correlation between the splenic weight and the platelet gain (p= 0.0568) and the splenic volume on preoperative imaging and the platelet gain (p= 0.0251).

**Conclusion:**

Early ligation of the splenic artery during splenectomy results in passive splenic decongestion and thereby a significant gain in blood components. This protocol appears to be a feasible blood conservation method to avoid blood transfusions in this group of hypersplenic EHPVO patients.

## INTRODUCTION

Extra hepatic portal venous obstruction (EHPVO) is common in the developing countries accounting for 70% of pediatric patients with portal hypertension and is second only to cirrhosis in the West. It is also the most common cause of upper gastrointestinal bleeding in children.[1] In congestive splenomegaly, the spleen volume can be 10 times greater than normal, with 30-90% of platelets and 40% of erythrocytes located in the spleen.[2–6] In children with EHPVO, splenorenal shunt after splenectomy is one of the procedures performed for the treatment of EHPVO with hypersplenism.[7] It is generally accepted that the spleen has the ability to autotransfuse a large quantity of red blood cells (RBC) into the systemic circulation during times of stress by contracting, thereby augmenting the blood's oxygen transport and increasing the blood components.[8]

The aim of the study was to objectively demonstrate this gain in blood components.

This study was based on the hypothesis that “early ligation of the splenic artery during splenectomy will result in decongesting the spleen prior to its removal thereby allowing a gain of a significant amount of sequestered blood components.”

## MATERIALS AND METHODS

This was a prospective study from 2007 to 2008. Patients diagnosed with EHPVO and hypersplenism and planned for splenectomy with portosystemic shunting were recruited into the study. Detailed history and clinical examination of patients was done. Clearance from the Institutional Ethics committee and informed consent of the patient/ parents were obtained. We followed a protocol of systematically locating and ligating the splenic artery first, followed by a short waiting period of 30 minutes to allow the massive spleen to decongest via the splenic vein and venous collaterals and then completing the splenectomy. Apart from this, standard steps for splenectomy were followed.

A blood sample (4 ml) was drawn just prior to splenic artery ligation for the estimation of radioactivity, hemoglobin, hematocrit, red blood cell count, red cell indices, total and differential leukocyte count, platelets, plasma protein, serum albumin, and globulin. A minimum period of 30 minutes was allowed to lapse, thereby allowing decongestion of the spleen via the splenic vein and collaterals. No intravenous fluid was administered during this 30-minute period. After 30 minutes, the splenic vein and collaterals were ligated and spleen was removed. Another blood sample (4 ml) was collected without stasis to reassess all the parameters. Splenic weight was measured soon after resection and the degree of splenomegaly was expressed as the splenic index (splenic weight/body weight).

Exclusion criteria were as follows:

1. Patients requiring administration of intravenous fluids or blood during the waiting period of 30 minutes following splenic artery ligation, and
2. Patients with a significant (more than 5 ml/kg body weight) amount of blood loss during the 30-minute waiting period.

Statistical analysis was carried out using STATA 9.0 (College station, Texas, USA). Data were presented as mean (±SD)/median (range) or number (percentage) as appropriate. Changes in hematological and biochemical parameters prior to splenic arterial ligation and after splenic decongestion were assessed using paired *t* test/Wilcoxon signed rank test. The correlation between splenic weight, splenic volume, and baseline hematological parameters and change in hematological parameters were measured using Spearman rank correlation coefficient. A *p* value of <0.05 was considered statistically significant.

## RESULTS

A total of 28 (20 male, 8 female) patients were studied. Splenectomy with splenorenal shunt was done in 26 cases, while splenectomy with inferior mesocaval shunt was done in 1 case, and splenectomy with right gastroepiploic-renal shunt was done in 1 case. The mean age at surgery was 9.9 (±3.2) years.

### Anthropometry and splenic measurements

The mean (SD) weight of patients was 28 (±10.3) kg and the mean body surface area (SD) was 1 (±0.23) m^2^. The splenic volume calculated on preoperative imaging studies (CECT/ MRI) ranged from 150 to 1905 cc with a median of 1057 cc, while the weight of the spleen specimen ranged from 145 to 1600 g with a median of 730 g. Median splenic index was 0.025 (range 0.01–0.084) and the median estimated intraoperative blood loss was 125 ml (range 30–800).

### Baseline hematological counts

All 28 patients had hypersplenism with varying combinations of anemia, leucopenia, and thrombocytopenia. The average baseline hematological counts have been shown in Table 1.

**Table 1:** Baseline Hematological Counts

Preoperative value (n =28)	Mean ± SD	Median (range)
Hemoglobin (g/dl)	9.99 ± 2.24	10.15 (4.6–16.1)
Hematocrit (%)	30.04 ± 6.61	30.5 (13–47)
Total leukocyte count (per mm^3^)	3907.69 ± 3224.27	2900 (1400–17900)
Platelet count (per mm^3^)	88242.31± 52072.24	68000 (17300–76000)

### Correlation of splenic weight and baseline hematological counts

**Figure 1:**
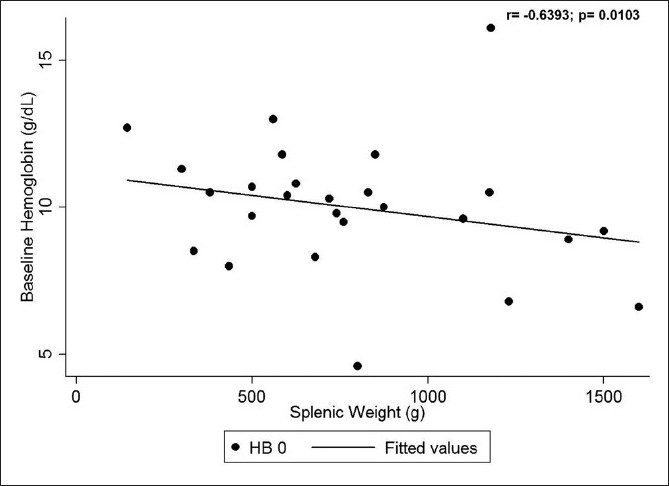
Correlation between splenic weight and baseline hemoglobin

**Figure 2:**
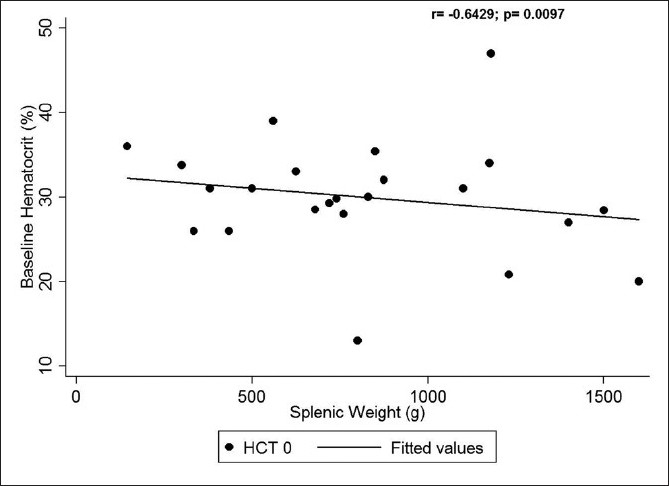
Correlation between splenic weight and baseline hematocrit

Our study also showed a significant negative correlation between the splenic weight and the baseline hemoglobin (Figures 1 and 2) (*r* = −0.6393, *p* =0.0103) and hematocrit (*r* =−0.6429, *p* = 0.0097) values (i.e., the larger the spleen, the lower were the baseline hemoglobin and hematocrit). However, no statistically significant correlation could be seen between the splenic weight and baseline total leukocyte and platelet counts.

### Hematological parameters

**Figure 3:**
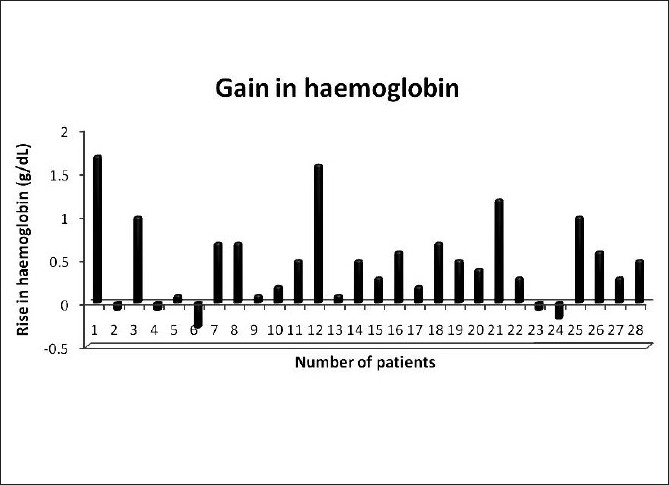
Pre-arterial ligation and post-splenic decongestion changes in hemoglobin

**Figure 4:**
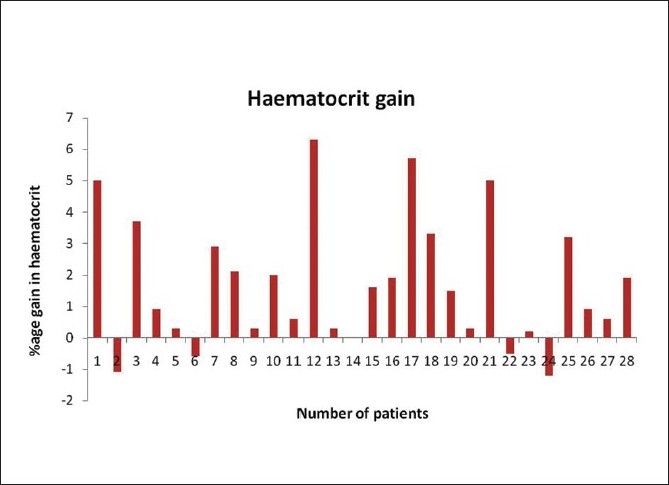
Pre-arterial ligation and post-splenic decongestion changes in hematocrit

The average post-splenic decongestion hematological parameters were significantly higher than the pre-arterial ligation values as shown in Table 2 and Figure 3 and 4.

**Table 2:** Hematological Counts

Variable (n=28)	(Mean ± SD)		p-value
		
	Prearterial ligation	Post-splenic decongestion	
Hemoglobin (g/dl)	10.99 ± 2.06	11.45 ± 2.03	<0.0001
Hematocrit (%)	36.85 ± 5.44	38.53 ± 5.74	0.0002
Total leukocyte count (TLC) (per mm^3^)	7987.5 ± 2911.14	10937.41 ± 3780.1	0.0001
Platelet count (per mm^3^)^a^	73000	92500	<0.0001
	(17000-183000)	(25000-216000)	
RBC count (×106 per mm^3^)	4.32 ±0.74	4.57 ± 0.7	0.0004

a Data presented as median (range).

### Change in hematologic parameters

**Figure 5:**
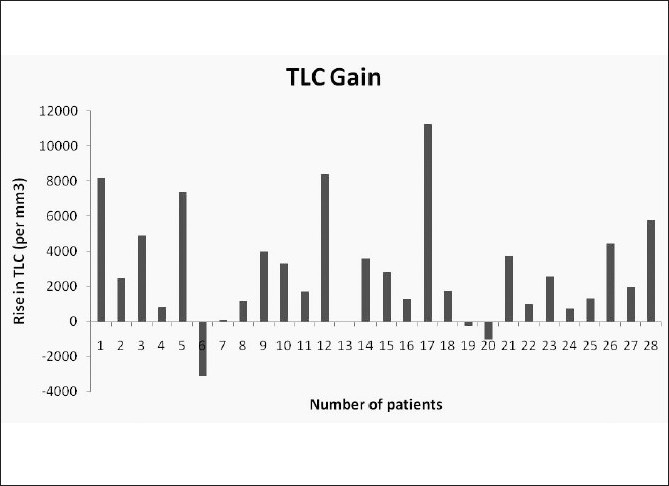
Pre-arterial ligation and post-splenic decongestion changes in TLC

Our study group showed a median rise in hemoglobin of 0.45 g/dl and a median rise in hematocrit of 1.2%. Similarly, the median rise in TLC Figure 5 and platelet counts were 2500/mm^3^ and 19500/mm^3^ respectively. The median rise in RBC count was 0.22×10^6^/mm^3^. The detailed results are shown in Table 3.

**Table 3:** Change in Hematologic Parameters

Gain/Loss (n= 28)	Mean ± SD	Median (range)
Hemoglobin (g/dl)	0.46 ± 0.49	0.45 (−0.3–1.7)
Hematocrit (%)	1.68 ± 2.04	1.2 (−1.2–6.3)
Total leukocyte count (per mm^3^)	2976.29 ± 3160.76	2500 (−3100–11270)
Platelet count (per mm^3^)	24817.86 ± 22241	19500 (−16000–76000)
RBC count (×106 per mm^3^)	0.19 ± 0.23	0.22 (−0.25–0.64)

### Correlation of gain in hematological counts with splenic size

**Figure 6:**
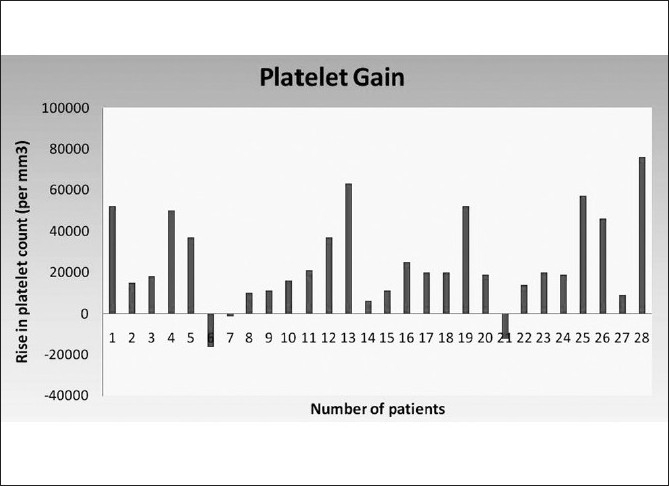
Pre-arterial ligation and post-splenic decongestion changes in platelet counts

**Figure 7:**
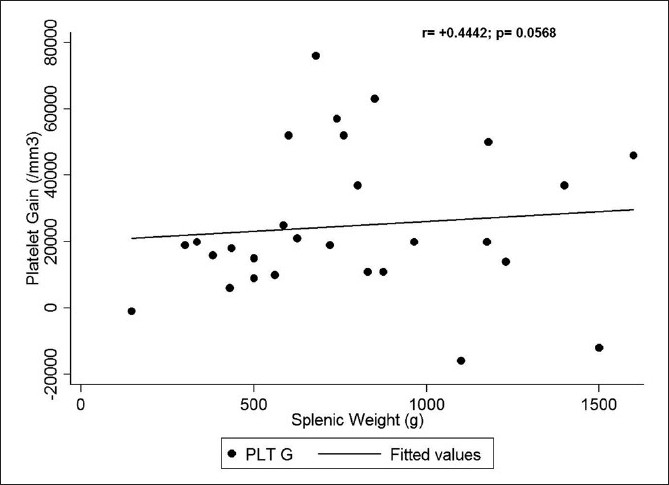
Correlation between splenic weight and platelet gain

**Figure 8:**
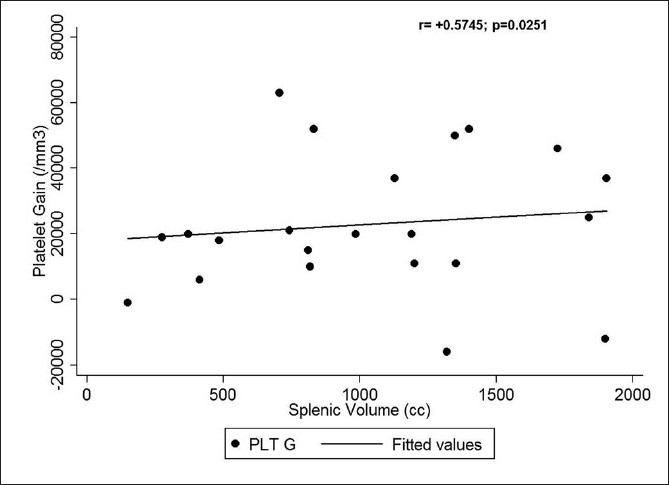
Correlation between splenic volume and baseline hemoglobin

A significant positive correlation was seen between the gain in platelet counts with both the preoperative splenic volume on imaging [Figure 6] (*r*=+0.5745, *p*=0.0251) and also the splenic [Figure 7] weight (*r*=+0.4442, *p*=0.0568). But no statistically significant correlation could be seen between the splenic weight or volume and the gain in hemoglobin, hematocrit, [Figure 8] or TLC.

There was no significant difference between the pre-arterial ligation and post-splenic decongestion RBC indices (*p*> 0.05). Similarly, there was no statistically significant difference between the biochemical parameters (total protein, serum albumin and globulins, blood urea, and serum creatinine) prior to splenic artery ligation and after splenic decongestion.

## DISCUSSION

The presence of hypersplenism in EHPVO patients frequently calls for RBC or platelet transfusion prior to or during surgery. Whether the spleen's ability to autotransfuse can be made use of prior to splenectomy to avoid allogenic blood transfusion in these hypersplenic patients is what we attempted to test by our study. We tested our hypothesis in 28 consecutive patients of EHPVO and analyzed the results of the same.

### Splenic size and hypersplenism

Jandl *et al.* and Cavalli *et al.* stated that in congestive splenomegaly, the spleen volume can be 10 times greater than normal, with 30–90% of platelets and 40% of erythrocytes located in the spleen.[9,10] Majority of children in our series also had massive splenomegaly with a median weight of 730 g and the median splenic volume on preoperative imaging studies was 1057 cc. The median splenic index was about 14 times higher than that reported in normal adult controls. Hypersplenism has been reported in 40–80% of EHPVO patients.[2,3] Shah *et al.* showed a negative correlation between splenic volume on USG and leukocyte counts.[11] Likewise, our study also showed a negative correlation between the splenic weight and the baseline hemoglobin and hematocrit values. But no statistically significant correlation could be seen between the splenic weight and baseline total leukocyte and platelet counts.

### Changes in hematologic parameters and equivalence to transfusion

We noticed a highly significant increase in the hemoglobin, hematocrit, TLC, platelet, and RBC counts by allowing the spleen to decongest and autotransfuse for at least 30 minutes. The median gain in hemoglobin by following our protocol was 0.45 g/dl, which is equivalent to a transfusion of at least half a unit (∼100–150 ml) of packed RBC.[12] The median rise in hematocrit of 1.2% can likewise be translated to a similar amount of packed RBC. There was also a significant rise in TLC with a median of 2500 cell/mm^3^. A platelet concentrate produced from a unit of whole blood contains, on average, 7.5 × 10^10^ platelets and has been shown to increase the platelet count by 5 to 10 × 10^9^/l (5,000–10,000/mm^3^) in a 70-kg recipient.[13] So the median rise in platelet count of 19,500/mm^3^ in our patients would translate to a platelet transfusion of at least 4 units of platelet concentrates in an adult. There was also a significant increase in the RBC count with a median rise of 0.22 × 10^6^/ mm^3^. Our study also showed a statistically significant positive correlation between the splenic weight and the platelet gain, and the splenic volume on preoperative imaging and the platelet gain, which can be extrapolated that the larger the spleen, the more would be the gain in blood components by following our protocol.

These results are encouraging, support our hypothesis, and may altogether help us to avoid allogeneic transfusions in these patients. Although we have no similar studies for comparison, we can cite two studies reporting effects of splenic contraction in humans. Espersen *et al.* have elicited modest but significant increases in hemoglobin concentration (0.64–1.93 g/dl) and hematocrit (0.3–1%) by interventions such as simulated diving and maximal apnea to induce splenic contraction in normal humans with intact spleens.[14] Baković *et al.* also did a similar study to calculate the contribution of splenic contraction to changes in the circulating volume of RBCs as well as the venous concentration of leucocytes and platelets following repeated breath-hold apneas in trained divers, normal controls, and splenectomized patients. The RBC volume and venous concentration of leucocytes increased in both trained apnea divers (4.9% and 14.9%, respectively) and intact subjects (1.7% and 7.2%, respectively), whereas in splenectomized subjects there was no change in RBC volume. However in this study, none of the groups showed significant changes in platelet concentrations.[8]

But unlike these reports, we did not use any intervention to actively induce splenic contraction. We merely allowed a waiting period for passive splenic decongestion after interrupting the splenic inflow. Whether using additional interventions like injectable epinephrine or manually compressing the spleen will further increase the gain in blood components from the spleen and the side-effects of such interventions remains to be elucidated.

As these patients only suffered from a congestive splenomegaly with no intrinsic defects in the RBC, we did not expect any changes in the red cell indices. The same was demonstrated by our study with no significant change in the MCV, MCH, and MCHC prior to and after splenic decongestion. These findings also allay any unwarranted fear about transfusing RBC trapped in the spleen into the peripheral circulation.

### Changes in biochemical parameters

While studying the effects of splenic contraction, Bakovic *et al.* also demonstrated that the plasma protein concentration decreased by 5.8% and 2% in apnea divers and untrained subjects, respectively, while the albumin concentration showed a significant decrease by 5.6% and 2.4%, respectively, indicating an expansion of plasma volume.[8] But in the study by Espersen *et al.*, the plasma protein concentration remained unchanged.[14] With this background, we also estimated biochemical parameters in our patients also prior to and after splenic decongestion. However, there was no significant change in these values. This result may be construed that the passive splenic decongestion led to a significant efflux of blood components but not so much of blood volume. Gain in red cells and platelets and consequently an improvement in the oxygen-carrying capacity of blood and platelet function without hemodilution may actually prove to be in the surgeons' and anesthetists' favor. This is again an interesting fact, which needs further clarification.

Children face the same risks of transfusion as adult patients.[15] Our method of utilizing passive splenic decongestion as a means of autologous blood transfusion appears to be a feasible blood conservation method in this group of hypersplenic EHPVO patients. Just by adhering to a simple protocol of sequential ligation of the splenic artery and allowing the massive spleen to decongest has helped us avoid allogeneic transfusions in our patients. Certain limitations were our inability to estimate the exact gain in RBC volume and to correlate the preoperative and intraoperative splenic volumetry. Nonetheless, our study has generated certain queries: (1) whether it is possible to predict in which patients this maneuver may be more beneficial by a preoperative splenic volume estimation, (2) can additional interventions to cause active splenic contraction aid further? and (3) can the same protocol be applied to patients undergoing splenectomy for splenomegaly due to other causes? These questions can form the basis for further studies.

## CONCLUSION

Early ligation of the splenic artery during splenectomy results in passive splenic decongestion and thereby a significant gain in blood components. This protocol appears to be a feasible blood conservation method to avoid allogeneic blood transfusions in this group of hypersplenic EHPVO patients.
